# plmmr: an R package to fit penalized linear mixed models for genome-wide association data with complex correlation structure

**DOI:** 10.1093/bib/bbaf672

**Published:** 2026-01-31

**Authors:** Tabitha K Peter, Anna C Reisetter, Yujing Lu, Oscar A Rysavy, Patrick J Breheny

**Affiliations:** Department of Biostatistics, University of Iowa, 145 N Riverside Dr, Iowa 52242, United States; Department of Biostatistics, University of Iowa, 145 N Riverside Dr, Iowa 52242, United States; Department of Biostatistics, University of Iowa, 145 N Riverside Dr, Iowa 52242, United States; Department of Biostatistics, University of Iowa, 145 N Riverside Dr, Iowa 52242, United States; Department of Biostatistics, University of Iowa, 145 N Riverside Dr, Iowa 52242, United States

**Keywords:** R, linear mixed models, lasso, penalized regression, statistical genetics

## Abstract

Correlation among the observations in high-dimensional regression modeling can be a major source of confounding. We present a new open-source package, **plmmr**, to implement **p**enalized **l**inear **m**ixed **m**odels in **R**. This R package estimates correlation among observations in high-dimensional data and uses those estimates to improve prediction with the best linear unbiased predictor. The package uses memory mapping so that genome-scale data can be analyzed on ordinary machines even if the size of data exceeds random-access memory. We present here the methods, workflow, and file-backing approach upon which **plmmr** is built, and we demonstrate its computational capabilities with two examples from real genome-wide association studies data.

## Introduction

Regression models for high-dimensional data have largely focused on independent observations, but correlation among samples can arise for many reasons, such as batch effects, geographic differences, ancestral groups, and/or family relationships. Such correlation can be a major source of confounding in data analysis. As a result, many approaches involve restricting the analysis to smaller groups of independent subjects. We present a new open-source package for high-dimensional regression capable of accounting for this correlation, thereby allowing the analysis to proceed incorporating data from all observations. Our package, **plmmr** (https://github.com/pbreheny/plmmr), implements **p**enalized **l**inear **m**ixed **m**odels in **R**. Of note, **plmmr** can handle latent/cryptic correlation structure (i.e. one does not need to know batch assignments or pedigree information), and scales up efficiently to handle genome-scale data such as genome-wide association studies (GWAS), even if the size of the data exceeds the memory of the machine.

Increasingly, batch effects have been recognized as having critical impacts on high-dimensional data [1]. One approach to addressing this type of correlation is to derive additional covariates in the form of principal components (PCs) or surrogate variables (SVs) and include them in the analysis [2, 3], although there is an inherent challenge in determining how many PCs/SVs to include in the model. This type of correlation is increasingly common in the context of human genetics due to an emphasis on increasing the diversity in GWAS data by intentionally recruiting participants from other ancestry groups [4]. Historically, most human genetics studies focused on homogeneous populations, with nearly 95% of existing GWAS data representing people of European ancestry [4].

While batch effects and population stratification result in large group structures, relational structures can also create small, highly correlated groups. An important case of this is family-based studies in GWAS, which have been acknowledged as valuable for the field [5]. At present, existing methodologies either assume that all family groups have the same known composition (e.g. all trios), or attempt to satisfy the assumption of independence by restricting the analysis to a set of unrelated individuals. However, identifying the largest subgroup of unrelated people in a given dataset is both an NP-hard problem and by definition results in excluding data from the analysis [6–9].

Large-scale and small-scale relationships among observations are often present in the same data set, such as a GWAS containing family groups from different geographic regions. Such combinations of relationships result in complex correlation structures. Furthermore, it is typically unrealistic to assume full knowledge of this structure—batch effects are usually not apparent, ancestry is complicated, and relationships may be cryptic. We describe in Section Preconditioning a linear mixed model the technique **plmmr** uses to accommodate complex correlation structures without requiring the relationships among observations to be known in advance.

An important distinction between **plmmr** and many other software packages that implement LMMs for high-dimensional data is that **plmmr** takes a joint modeling approach as opposed to a one-at-a-time (or “marginal”) approach. A joint modeling approach is an additive model that considers the cumulative impact of all features in the data. A joint model identifies important features via sparsity-inducing penalties, such that the final model includes only the features that improve prediction of the phenotype. As such, one advantage of the joint modeling approach over such a marginal approach is that in the former, we directly construct a predictive model. This has implications for polygenic risk score calculation, as polygenic risk scores based on one-at-a-time testing require additional steps to combine multiple marginal models into a single prediction. Recognizing this advantage of joint modeling, several recent approaches (e.g. BOLT-LMM [10], SAIGE [11], fastGWA [12], and REGENIE [13]) use a two-step approach in which a joint model is used as a first step. The joint modeling step is then followed by marginal testing designed to produce per-variant results. Our **plmmr** package offers something new as it implements a joint model in one single step—results are provided from the regression model, instead of having a second step of marginal testing.

Our presentation of the **plmmr** package is organized as follows: Section Implementation summarizes the methodological approach for handling correlation, outlines the workflow of the **plmmr** pipeline, and describes the file-backing technique **plmmr** uses to scale up to large data. Section Results presents computational time for **plmmr** analyses using real GWAS data. Finally, Section Discussion situates **plmmr** in the current landscape of tools available for analyzing correlated GWAS data, outlining strengths, limitations, and future directions for our proposed approach.

## Methods

### Preconditioning a linear mixed model

In order to incorporate complex correlation structure into the model for the data, **plmmr** uses a technique that projects the data onto a transformed scale. This technique has been called “preconditioning” in the literature—e.g. see [14] or [15]. In brief, preconditioning requires a preconditioning matrix (the “preconditioner”) $\mathbf{F}$ and transforms the problem $\mathbf{y} = \mathbf{X}\boldsymbol{\beta }$ into $\mathbf{F}\mathbf{y} = (\mathbf{F}\mathbf{X})\boldsymbol{\beta }$. In our model, we define $\mathbf{X} = n \times p$ as a standardized design matrix, and $\mathbf{y} = n \times 1$ as the outcome of interest. We then define $\mathbf{K} = \frac{1}{p}\mathbf{X}\mathbf{X}^{\scriptscriptstyle \top }$. Note that in the specific context of GWAS where $\mathbf{X}$ is a genotype matrix, $\mathbf{y}$ is the phenotype and $\mathbf{K}$ is known as the genomic relatedness matrix (GRM, also known as the “kinship” matrix as defined by [16]). We then adopt the linear mixed model proposed by [17]:


(1) \begin{align*}& \mathbf{y} = \mathbf{X}\boldsymbol{\beta} + \mathbf{u} + \boldsymbol{\epsilon}\end{align*}

where random effect $\mathbf{u}$ represents an **u**nobserved random effect with the distribution $\mathbf{u} \sim N(\mathbf{0}, \sigma ^{2}_{s}\mathbf{K})$. Under the standard assumptions that $\boldsymbol{\epsilon } \perp \mathbf{u}$ and $\boldsymbol{\epsilon } \sim N(0, \sigma ^{2}_\epsilon \mathbf{I})$, the variance of $\mathbf{y}$ may be written $\boldsymbol{\Sigma } = \sigma ^{2}_{s}\mathbf{K} + \sigma ^{2}_\epsilon \mathbf{I}$, with $\sigma ^{2}_{s}$ representing the variance of $\mathbf{y}$ due to population **s**tructure and $\sigma ^{2}_\epsilon $ represents the variation in $\mathbf{y}$ due to noise. Model (1) can therefore be equivalently written


(2) \begin{align*}& \mathbf{y} \sim N\big(\mathbf{X}\boldsymbol{\beta}, \sigma^{2}_{s}\mathbf{K} + \sigma^{2}_\epsilon\mathbf{I}\big) \equiv \mathbf{y} \sim N(\mathbf{X}\boldsymbol{\beta}, \boldsymbol{\Sigma}).\end{align*}

We precondition Equation (2) using $\mathbf{F}$, where $\boldsymbol{\Sigma } = \mathbf{F}\mathbf{F}^{\scriptscriptstyle \top }$, to obtain


(3) \begin{align*}& \mathbf{F}\mathbf{y} \sim N((\mathbf{F}\mathbf{X})\boldsymbol{\beta}, \mathbf{I}),\end{align*}

which we re-express as


(4) \begin{align*}& \tilde{\mathbf{y}} \sim N(\tilde{\mathbf{X}}\boldsymbol{\beta}, \mathbf{I}),\end{align*}

where $\tilde{\mathbf{X}}$ and $\tilde{\mathbf{y}}$ represent the design matrix and outcome vector on the rotated scale, respectively. As shown in Equation (4), this preconditioning serves to “decorrelate” the variance structure so that observations on the $\tilde{\mathbf{y}}$, $\tilde{\mathbf{X}}$ scale are independent. [18] evaluated this modeling framework in the specific application to genetics studies with confounding.

In (4), it is important to note that the meaning of the features and their coefficients is unchanged. In other words, $\beta _{j}$ still represents the effect of feature $j$, even though row $i$ of $\tilde{\mathbf{X}}$ no longer corresponds to subject $i$. This is the opposite of what happens in a transformation such as PCs that operates on the columns of $\mathbf{X}$ instead of the rows ($\mathbf{X}\mathbf{F}$ instead of $\mathbf{F}\mathbf{X}$). In a PCs analysis, $\beta _{j}$ no longer corresponds to feature $j$, although row $i$ still corresponds to subject $i$.

At present, the **plmmr** package uses $\mathbf{K} = \frac{1}{p}\mathbf{X}\mathbf{X}^{\scriptscriptstyle \top }$, although other estimates of the relatedness matrix have also been proposed and may be incorporated in the future. In particular, sparse estimates of $\mathbf{K}$ have also been proposed, and offer potential computational advantages [19, 20]. Other preconditioning matrices that manipulate the eigenvalues of $\mathbf{K}$ have also been proposed [14, 21].

### Penalizing the mixed model

On the transformed scale expressed in (4), existing regression approaches may be applied. For example, if $\boldsymbol{\beta }$ consists of a small number of coefficients, classical linear regression could be used. This occurs often, for example, if one is fitting a large number of marginal models for each single-nucleotide polymorphism (SNP) or each gene separately. However, if one wishes to construct a joint regression model involving all of the biological features at the same time, some form of penalization is required in order to deal with the large number of parameters. In particular, *penalized linear mixed models* estimate $\boldsymbol{\beta }$ by minimizing the objective


\begin{align*} & \frac{1}{2n} \lVert\tilde{\mathbf{y}} - \tilde{\mathbf{X}}\boldsymbol{\beta}\rVert_2^2 + P_\lambda(\boldsymbol{\beta}), \end{align*}

where $\lVert \tilde{\mathbf{y}} - \tilde{\mathbf{X}}\boldsymbol{\beta }\rVert _{2}^{2}$ is the residual sum of squares objective from classical linear regression, $P$ is a penalty function that penalizes large values of $\boldsymbol{\beta }$, and $\lambda $ is a regularization parameter that controls the tradeoff between the two components.

A widely used penalty is the lasso [22], where $P_\lambda (\boldsymbol{\beta }) = \lambda \sum _{j=1}^{p} \left \lvert \beta _{j}\right \rvert $ penalizes the absolute values of the coefficients. An important reason for its popularity is that the solutions are sparse, meaning that many coefficients are exactly zero and only the most relevant predictors appear in the model. Other penalties, such as SCAD [23] and MCP [24], introduce less bias than the lasso (at the cost of increased variance), while the elastic net [25] is more stable in the presence of highly correlated predictors.

### Prediction

Prediction plays a central role in modeling for two key reasons. First, it is essential for model selection—in this context, for choosing the regularization parameter $\lambda $—which in turn determines which features are selected. Second, prediction is also of direct importance for a wide range of applications, including forecasting clinical outcomes such as heart disease, guiding plant and animal breeding, constructing polygenic risk scores, and conducting causal inference through Mendelian randomization.

Best linear unbiased prediction (BLUP) incorporates the correlation/relationship between outcomes in addition to the direct effects of individual features, and this approach increases accuracy in a wide variety of applications [26]. Let $\{\mathbf{X}_{1}, \mathbf{y}_{1}\}$ represent the data used to fit a penalized linear mixed model, $\mathbf{X}_{2}$ represent new data for which predictions are to be made, and partition $\boldsymbol{\Sigma } = \mathbb{V}(\mathbf{y}_{1}, \mathbf{y}_{2})$ as


\begin{align*} & \hat{\boldsymbol{\Sigma}} = \begin{bmatrix} \hat{\boldsymbol{\Sigma}}_{11} && \hat{\boldsymbol{\Sigma}}_{12} \\ \hat{\boldsymbol{\Sigma}}_{21} && \hat{\boldsymbol{\Sigma}}_{22} \end{bmatrix}. \end{align*}

Note that $\hat{\boldsymbol{\Sigma }}_{11}$ has already been calculated and used in the preconditioning (2) and (3). Furthermore, the sample correlation between $\mathbf{X}_{1}$ and $\mathbf{X}_{2}$ provides an estimate for $\hat{\boldsymbol{\Sigma }}_{21} = \tfrac{1}{p}\mathbf{X}_{2}\mathbf{X}_{1}^{\scriptscriptstyle \top }$. Using these quantities, the BLUP for the new outcome $\mathbf{y}_{2}$ is


\begin{align*} & \hat{\mathbf{y}}_2 = \mathbf{X}_2\widehat{\boldsymbol{\beta}} + \hat{\boldsymbol{\Sigma}}_{21}\hat{\boldsymbol{\Sigma}}_{11}^{-1}(\mathbf{y}_1 - \mathbf{X}\widehat{\boldsymbol{\beta}}). \end{align*}

Note that the first part of this equation is the standard linear predictor from linear regression, while the second term adjusts this prediction based on the similarity between subjects in the original and new data sets. This adjustment allows the model to explain part of the outcome through correlation among observations, reducing the burden on the linear predictor. As a result, fewer features need to be selected, leading to sparser solutions. This not only improves predictive accuracy, but also helps mitigate confounding and reduce false positives by properly accounting for the correlation structure in the data.

### Workflow: from data files to model results

With current available tools, carrying out the analysis described in 2.1 requires a variety of different software packages written in different languages. Users must link together these various tools, typically using command-line functions. Requiring each analyst to code their own pipeline is inefficient, error-prone, and presents a barrier to reproducibility.

**Figure 1:**
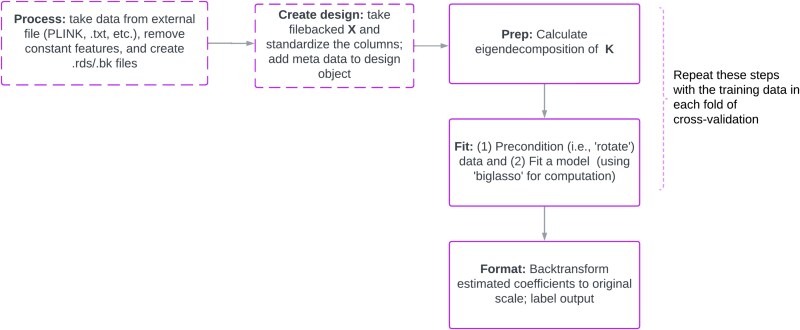
Workflow for plmmr. Steps shown with dotted lines are optional; steps shown with solid lines indicate essential components of the workflow.


This motivated us to create **plmmr**, which offers an integrated workflow as shown in Fig. 1. In its simplest form, if the user has already processed the features and outcome, one can fit the penalized linear mixed model with

\begin{align*} \text{plmm(x, y)} \end{align*}

which carries out all the steps on the right-hand-side of Fig. 1.

A more complicated example workflow, where the data is stored in PLINK files on the hard drive and must be integrated with data in a separate file storing phenotype data, is given below. Since all steps use the same R package, there is no need to convert between file types, data structures, programming languages, etc.

# assuming that files plink.bed/plink.bim/plink.fam

# are stored in directory "data_dir":

library(plmmr)

# create filebacked object from PLINK data files

plink_data <- process_plink(data_dir = "data_dir",

$ \qquad \qquad \qquad \qquad \qquad \qquad \qquad \mathtt{data}\_\mathtt{prefix} \ = \ \mathtt{"plink"},$

$ \qquad \qquad \qquad \qquad \qquad \qquad \qquad \mathtt{rds}\_\mathtt{dir} \ = \ \mathtt{"some}\_\mathtt{dir"},$

$ \qquad \qquad \qquad \qquad \qquad \qquad \qquad \mathtt{rds}\_\mathtt{prefix} \ = \ \mathtt{"plink}\_\mathtt{data"})$

# read in phenotype data

phen <- read.csv("clinical.csv")

# create a design

design <- create_design(data_file = plink_data,

$ \qquad \qquad \qquad \qquad \qquad \qquad \mathtt{feature}\_\mathtt{id} \ = \ \mathtt{"FID"},$

$ \qquad \qquad \qquad \qquad \qquad \qquad \mathtt{rds}\_\mathtt{dir} \ = \ \mathtt{"some}\_\mathtt{dir"},$

$ \qquad \qquad \qquad \qquad \qquad \qquad \mathtt{ new}\_\mathtt{file} \ = \ \mathtt{"design"},$

$ \qquad \qquad \qquad \qquad \qquad \qquad \mathtt{add}\_\mathtt{outcome} \ = \ \mathtt{phen,}$

$ \qquad \qquad \qquad \qquad \qquad \qquad \mathtt{outcome}\_\mathtt{id} \ = \ \mathtt{"ID",}$

$ \qquad \qquad \qquad \qquad \qquad \qquad \mathtt{outcome}\_\mathtt{col}\ = \ \mathtt{"outcome")}$

# fit a model

fit <- plmm(design)

# summarize coefficients at 50th lambda value

summary(fit, idx = 50)

# plot of estimated coefficient paths

plot(fit)

The first step in the workflow above involves creating an R object corresponding to the input data. **plmmr** is designed to accept multiple forms of data input, including a delimited file or a set of PLINK files. For data coming from external files too large to read into memory, the **plmmr** workflow includes a processing step that creates a pointer object to the external file(s) rather than reading them into R. This filebacking approach (described in greater detail in Section Filebacking and integration with C++) allows **plmmr** to analyze GWAS-scale data even on machines where memory is limited.

Once there is an R object representing the data, create_design() takes this object as input and implements the following measures to prepare data for model fitting:

1. Integrates outcome (e.g. phenotypic) information
2. Option: designate additional, unpenalized features
3. Standardizes design matrix $\mathbf{X}$

Integrating the outcome information into the design is not necessarily trivial, as merging $\mathbf{X}$ and $\mathbf{y}$ requires proper alignment with respect to the order of the observations; create_design() checks for this alignment, rather than assuming the user has already addressed this issue. The create_design() function also has several options for designating unpenalized features; for GWAS data, features from another file such as age and sex may be merged in with the genotype data as unpenalized covariates in the model design. The final design matrix is column-standardized, and it is returned as part of the plmm_design object returned by create_design(). This object can be passed directly into the main model fitting function plmm().

The internal work of the plmm() function is made up of three steps, which we refer to as (i) the “prep” step, (ii) the “fit” step, and (iii) the “format” step. The “prep” step prepares the preconditioning matrix to be used in analysis by taking an eigendecomposition of the matrix $\mathbf{K}$. The eigendecomposition of $\mathbf{K}$ is necessary for constructing the preconditioning matrix $\boldsymbol{\Sigma }^{-1/2}$. The fit step uses a coordinate descent algorithm to fit the model. The “format” step transforms the estimated coefficients back onto the scale of the original data – this is done for clarity of interpretation. The results of plmm() can be passed directly into **plmmr**’s plot() and summary() methods, so that there is seamless integration with simple syntax throughout the entire workflow.

plmm() is designed to be flexible to the needs of the user, offering many optional arguments that allow the user to customize model fitting details such as the choice of $\lambda $. Importantly, the penalty argument specifies the type of penalty to be used in modeling, with options including lasso (the default), SCAD, or MCP. Elastic net penalization is also available via the optional alpha argument, analogous to the syntax for the mixture parameter in **glmnet** [27].

In addition to model fitting, **plmmr** also offers a cross-validation (CV) method, cv_plmm(), that both fits a model and chooses its tuning parameter $\lambda $ with the syntax shown below:

cv_fit <- cv_plmm(design)

# plot and summary methods:

summary(cv_fit)

plot(cv_fit)

Care must be taken when applying CV to the analytical approach of 2.1, as preconditioning has implications for exchangeability. Although standard penalized regression software can be used to fit a model on preconditioned data, the CV methods these other software supply will be incorrect if the preconditioning step is not included in each cross-validation fold. Correct implementation of CV requires that every part of the model-fitting process be cross-validated [28]. A homebrewed pipeline is liable to get this part of the analysis wrong and lead to unintentional errors. We further developed these ideas in the methods work behind **plmmr** [29], so that the CV method in **plmmr** is integrated with the entire model-fitting process [30]. The cv_plmm() return value may be passed directly to plot() and summary() methods as well; example output from plot() is shown in Fig. 4. **plmmr** implements BLUP as the default in the predict() method, and BLUP is the default prediction method used in cv_plmm().

### Filebacking and integration with C++

One major challenge in analyzing GWAS-scale data is the limitation of random-access memory (RAM), which motivated the design of **plmmr** as a package that uses file-backing. In cases where $\mathbf{X}$ is too large for one machine’s RAM to accommodate, **plmmr** creates a file on disk, assigns a C++ pointer to this file, and allows that pointer to be accessible as an R object. The user then interacts with the pointer in the R session, so that the data are not read into memory. This technique of creating files on disk has been often employed to analyze large data [31, 32]. **plmmr** builds on the **bigmemory** package infrastructure for creating R objects that ‘‘point’’ to files on disk. The major model fitting steps use **bigalgebra** [33] and **biglasso** [34], operating in C++ on the data stored in the binary file. This improves computational time and ensures that the design matrix, $\mathbf{X}$, is never read into memory. The output from a plmm() model includes the estimated coefficients for each predictor at each value of the tuning parameter, saved in a sparse format as offered by the **Matrix** package [35]. In this way, the input to the model fitting function, the model fitting process itself, and the object returned are optimized to be memory-efficient and enable analyses to be run on a personal computer.

## Results

Analyzing real data is essential for demonstrating that software is scalable, accessible, and useful in practice. In this section, we illustrate the capabilities of the **plmmr** package in three scenarios, each defined by a real dataset. The first dataset is taken from a gene expression study and illustrates that the utility of **plmmr** is not limited to GWAS applications and can be used to address batch effects (Section Bladder cancer gene expression); the second dataset is a GWAS in which we carry out detailed timing comparisons and runtime breakdowns (Section Coronary artery disease GWAS); and the third dataset is from a much larger, multi-site, family-based GWAS that highlights the performance of **plmmr** in the presence of complex population stratification (Section Orofacial clefting GWAS). Across these real-world analytical scenarios, we demonstrate the **plmmr** methods for model building, cross-validation, prediction, and plotting.

To provide points of reference from the existing landscape of tools available for high-dimensional data analysis, we also draw some comparisons between the performance of **plmmr** and that of other open-source packages, including the R package **ggmix** [36], the R package **glmnet** [37], and the Julia package **PenalizedGLMM** [38].

### Bladder cancer gene expression

Dyrskjøt et al. [39] carried out a study of gene expression in patients with bladder cancer. Subsequent re-analyses of this data have shown that batch effects are a concern, as the presence or absence of cancer is confounded with the batch in which the samples were processed [1]. We analyzed this data with cancer as a 0/1 outcome and gene expression as predictors, using leave-one-out cross-validation for model selection. Prediction error is measured using the out-of-sample root mean square prediction error (RMSPE).

The **plmmr** model selected 23 genes with a RMSPE of 0.17. To evaluate the biological plausibility of these selections, we assembled a broad list of cancer-related genes from the MSigDB C2 collection whose names included either ‘‘cancer’’ or ‘‘tumor.’’ According to this criterion, 20/23 (87%) of **plmmr**’s genes were plausibly related to bladder cancer.

A regular lasso model (fit using **glmnet**) treating observations as independent selected 37 genes and resulted in a 29% higher prediction error (RMSPE = 0.22). Furthermore, only 81% of these genes selected fit our definition of plausible, suggesting that some of the genes selected by the lasso are false positives, and that this effect is mitigated by the use of a mixed model.

A different mixed model, fit using **ggmix**, produced a similar RMSPE (0.17), but selected considerably more genes: 51, of which 41 (80%) fit our definition of plausible. The two packages fit the same underlying model, but differ in terms of how they estimate the variance components $\sigma _{s}$ and $\sigma _{\epsilon }$.

### Coronary artery disease GWAS

**Figure 2:**
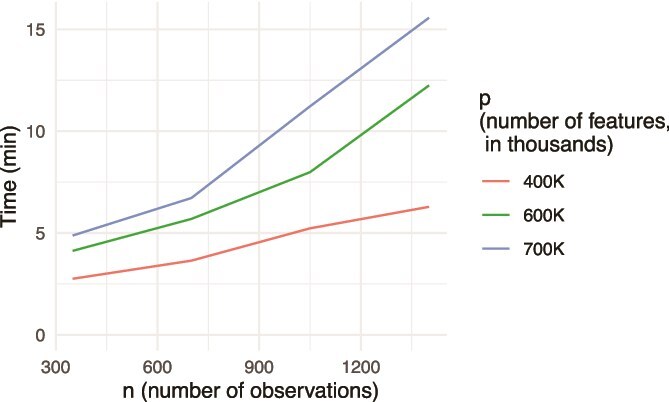
Total pipeline time in minutes: includes pre-processing, eigendecomposition, and model fitting.

The PennCath study [40] was a population-based GWAS of 1401 American participants of European ancestry in which the phenotype of interest was coronary artery disease. The genotype data for this study represent about 800000 SNPs, and these data have been made publicly available. Starting with these data, we used genotype data from 696644 autosomal SNPs that passed the quality control criteria (see Supplemental Material for quality control details) in order to illustrate the computational capabilities of **plmmr**. After quality control measures were taken, we created eleven subsets of genotype data using arbitrary filtering of samples and features. With eleven subsets and the full data set, we had twelve data sets of different dimensions. Among these twelve data sets, we varied the number of samples, $n$, so that $n \in \{350, 700, 1050, 1401\}$. We also varied the number of features, $p$, so that $p \in \{400\textrm{K}, 600\textrm{K}, 700\textrm{K}\}$ (where $\textrm{K} \equiv 1000$, and $p = 700\textrm{K}$ denotes the entire set of $696,646$ autosomal SNPs passing QC checks). Figure 2 reports total runtimes for each subset. Analyses were carried out on a Linux system running OpenBLAS [41] on an Intel Core i7-8700 CPU (3.20 GHz, 6 cores / 12 threads). Total time ranged from 2.5 min for the smallest subset ($n = 350, p = 400 \textrm{K}$) to 15 minutes for the full PennCath data ($n = 1401, p = 700\textrm{K}$).

**Figure 3:**
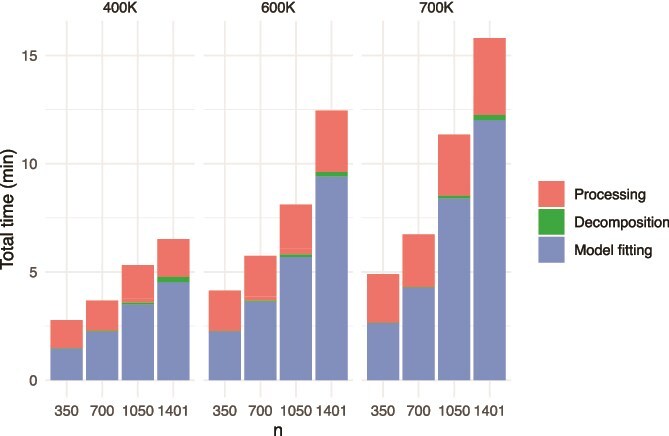
Time (in minutes) spent in each stage of the plmmr pipeline.

For every data subset, we also timed each step of the **plmmr** pipeline: processing the PLINK files with process_plink() and create_design(), decomposition of the covariance matrix, and fitting a penalized linear mixed model with lasso penalization via plmm(). These results are shown in Fig. 3. We found that the pre-processing never took longer than about 5 minutes, and the proportion of time spent in the actual optimization of the PLMM objective increased with $n$. These times do not include cross-validation, although CV does not necessarily increase computational time, as it can be run in parallel.

**Figure 4:**
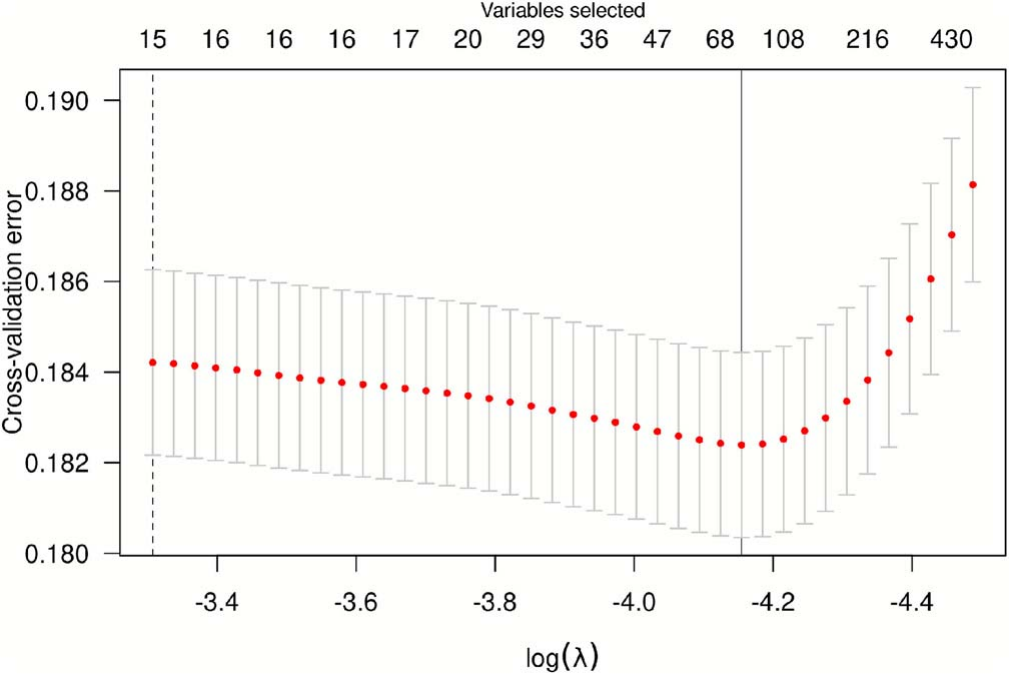
Plot of cross-validation error for plmmr models fit to the POFC dataset (*n* = 10 545, *p* = 467 577), using a sequence of 100 values of the regularization parameter, with the first 40 shown.

As this is a study of unrelated individuals in a relatively homogeneous population, we would not expect mixed models to provide a significant advantage in this scenario. Indeed, we obtained nearly identical results in terms of both prediction error and which features were selected whether using **plmmr**, **glmnet**, or **PenalizedGLMM**. To evaluate prediction error, we split the data into training and testing sets with $1000$ observations and $401$ observations, respectively. All methods achieved essentially the same RMSPE of 0.43, a clear improvement upon the null model RMSPE of 0.48. Both the **glmnet** and **plmmr** approaches selected the same four parameters, whilethe **PenalizedGLMM** approach selected five parameters, of which three (sex, age, and a single SNP) agreed with the other models. This is possibly due to the fact that the cross-validation method implemented within **PenalizedGLMM** is still under development at the time of writing, so we used the Bayesian Information Criterion to select $\lambda $ for this approach. Furthermore, all three approaches selected sex and age, which we included as positive controls due to their well-established relevance to coronary artery disease.

### Orofacial clefting GWAS

To illustrate **plmmr** at work with more complex correlation structures, we used the **plmmr** pipeline to analyze data from the Pittsburgh Orofacial Cleft (POFC) study as our third example. Additional information on the Pittsburgh Orofacial Cleft Study is available at https://www.dental.pitt.edu/research/ccdg/participate-research/pittsburgh-orofacial-cleft-studies. The POFC study was a global, family-based GWAS in which the phenotype of focus was orofacial cleft (e.g. cleft palate). The GWAS data from the POFC study represents $10\,545$ participants from over $2500$ families, and these families were recruited from fourteen global sites across five continents. The design matrix for this example included biological sex and country of recruitment site as unpenalized covariates, as these factors are known to be related to orofacial cleft formation [42]. While these genetic data were collected over ten years ago, **plmmr** has made it possible to include all of these participants (cleft patients, control patients, and all family members) in a single analysis for the first time.

A total of $469\,577$ SNPs remained in the analytical POFC data set after quality control measures were applied and SNPs with minor allele frequency below $0.0001$ were removed. As a proof of concept, this analysis was run in a container using a single core and a modest amount of memory. The total runtime was 31.6 h on a single Intel Xeon CPU @ 2.40 GHz. Selection of lasso tuning parameter $\lambda $ was done with five-fold cross-validation. Figure 2 shows the cross-validation error (CVE) across the first 40 candidate values of $\lambda $. At the left side of the plot, no SNPs are included; this is simply the classical BLUP with the design covariates included (sex, recruitment site). Including additional SNPs from the PLMM improves the prediction accuracy until we reach the optimal value of $\lambda $, at which point 62 SNPs are selected. A thorough analysis of these selected SNPs has been described elsewhere [43]; for the present paper, we highlight that the genes represented by these selected SNPs included several genes that have been identified as associated with orofacial clefts in previous literature, including: NTN1, PAX7, IRF6, DCAF4L2, EPHA3, and FOXE1 [44--46].

To explore an alternative approach for analyzing these data, we also used **glmnet** to select a model with five-fold cross-validation, using the same $\lambda $ values and the same unpenalized covariates. This **glmnet** analysis was run on a high-performance computer with 350 GiB of available RAM. The **glmnet** implementation selected a model with 67 SNPs, comparable to **plmmr** in terms of the model size. There was substantial overlap in the SNPs selected by the **plmmr** and **glmnet** models, with the two models identifying 51 of the same SNPS, implying 41 of the same genes; for example, both models identified SNPs on IRF6, PAX7, and NTN1. Of the 11 genes implied only by the **plmmr** model, we noticed that several—including EPHA3, FOXE1, and DCAF4L2—are genes that have been identified in previous literature as associated with nonsyndromic orofacial clefts. Of the 16 genes implicated only by the **glmnet** model, none of these correspond to genes known to be associated with nonsyndromic orofacial cleft. As in Section Bladder cancer gene expression, these results suggest that mixed models are effective at mitigating confounding and identifying features more likely to be have a true biological relationship with the outcome.

## Discussion

The **plmmr** package implements a joint mixed modeling approach for selecting features of interest while accounting for correlation. Several related tools have also been developed. Table 1 provides a succinct comparison of their capabilities at the time of writing, although the comparison should be interpreted with caution as many of these packages are actively adding new features.

**Table 1:** Comparison of currently available features in **plmmr** and similar packages

Software	Language	Penalties Implemented				PennCath Time (min)	Logistic Regression	Cross- Validation
		Lasso	Elastic Net	MCP	SCAD			
plmmr	R	$\bullet $	$\bullet $	$\bullet $	$\bullet $	11.6	$\times $	✓
ggmix	R	$\bullet $	$\bullet $			Crash	$\times $	$\times $
PenalizedGLMM	Julia	$\bullet $				13.8	✓	✓
glmmPen	R	$\bullet $	$\bullet $	$\bullet $	$\bullet $	N/A	✓	$\times $
HighDimMixedModels	Julia	$\bullet $			$\bullet $	N/A	$\times $	✓

PennCath time: records the time required to analyze the data set described in Section Coronary artery disease GWAS with the computing setup specified in that same section. Note that some packages cannot be applied to this type of data.

Both the **glmmPen** [47] and **HighDimMixedModels** [48] packages implement penalized mixed models, but these packages assume that the factors which govern the correlation between subjects are known, and cannot be directly applied to the setting in which these relationships must be inferred or estimated, as in population genetics. In addition, **glmmPen** currently lacks executable examples bundled within the package (an example script is available on the author’s personal site, although its ongoing maintenance is not clear).

Other packages that implement mixed models for correlated, high-dimensional data include the R package **ggmix** [49]. While **ggmix** uses a similar transformation technique as **plmmr**, **ggmix** does not scale up to large, genome-scale data as well as **plmmr**, and crashes when used to fit the GWAS data of Section Coronary artery disease GWAS. In addition, the package does not currently offer a cross-validation method (see Supplemental Material for further discussion of computational details).

To our knowledge, **plmmr** is the only package that enables fitting PLMMs on datasets that exceed RAM capacity, with a fully integrated workflow that includes BLUP-based cross-validation. Alongside this integrated workflow, **plmmr** offers thorough documentation including vignettes that users can work through using datasets bundled with the package. This documentation gives **plmmr** an accessibility that is not common among bioinformatics software.

One limitation of **plmmr** is that the required eigendecomposition of the relatedness matrix $\mathbf{K}$ becomes computationally expensive as $n$ grows. As we demonstrate, the approach remains feasible for datasets up to $n \approx 10,000$, though it is not currently suitable for biobank-scale data. To address this, we are investigating strategies to improve scalability with respect to $n$, including sparse matrix methods and approximate eigendecompositions [50].

Another limitation of **plmmr** is that it currently does not offer logistic regression. Binary outcomes can be analyzed, but they must be treated as numeric and analyzed with linear models. We are actively working to extend the penalized linear mixed modeling framework presented here to include logistic regression.

As mentioned in Section 3, [38] recently introduced a Julia package, **PenalizedGLMM**, for fitting PLMMs with inferred relationships between subjects, specializing in the case of PLINK-formatted SNP data. Although the package is not yet registered and its documentation is incomplete, **PenalizedGLMM** fills an important gap in the field by supporting logistic regression, a capability not currently available in any relationship matrix-based PLMM tool. Furthermore, we find its scalability and speed to be comparable to that of **plmmr**, although it lacks **plmmr**’s out-of-core capabilities. Given their complementary strengths, **PenalizedGLMM** and **plmmr** currently offer the most scalable and efficient tools for incorporating relationship matrices into high-dimensional penalized regression in Julia and R, respectively.

**Key Points**

- We have presented here a new R package, **plmmr**, which offers the capacity to fit penalized linear mixed models to genome-wide association studies-scale data with complex correlation structure.
- The software provides an end-to-end workflow that takes the user through all steps of the analysis in a single integrated pipeline, from processing raw data (e.g. PLINK files) to model summaries, including cross-validation.
- Calculations are carried out in C++ for computational efficiency.
- **plmmr** also provides an option to create and work with memory-mapped files, allowing the user to analyze large data sets that cannot fit in memory.

## Supplementary Material

supplement_bbaf672
