# Supplementary material for: plmmr: an R package to fit penalized linear mixed models for genome-wide association data with complex correlation structure
Source: Brief Bioinform. 2026 Jan 31;27(1):bbaf672. doi: 10.1093/bib/bbaf672 (PMC12860386; doi:10.1093/bib/bbaf672)
Supplement: supplement_bbaf672 [file supplement_bbaf672.pdf]

Supplemental material for "plmmr: an R package to fit penalized linear mixed models for genome-wide association data with complex correlation structure"

Tabitha K. Peter
Dept. of Biostatistics
University of Iowa

Anna C. Reisetter
Dept. of Biostatistics
University of Iowa

Yujing Lu
Dept. of Biostatistics
University of Iowa

Oscar A. Rysavy
Dept. of Biostatistics
University of Iowa

Patrick J. Breheny
Dept. of Biostatistics
University of Iowa

November 25, 2025

S1 Availability of data and materials

The **plmmr** package has been published on GitHub and made available on CRAN at `https://cran.r-project.org/web/packages/plmmr/index.html`. The package ships with three example datasets, one for each type of input: (1) data that is read into memory, (2) delimited file input, and (3) a set of PLINK files (.bed/.bim/.fam) input. The documentation website (`https://pbreheny.github.io/plmmr/`). includes tutorial-style articles with hands-on examples of how to analyze data from each of these formats. Users are able to work through the examples in these articles interactively using the datasets that are included with **plmmr**

installation.

All of the code presented in relation to the PennCath data example (as described in Section 3.2) has been made available in a public GitHub repository: https://github.com/tabpeter/reproduce_plmmr_manuscript. This public repository includes a link to the download for the published GWAS data, so that readers may download the PennCath GWAS data, clone the repository, install **plmmr**, and then reproduce the figures shown here on their own machines.

The GWAS data from the Pittsburgh Orofacial Cleft study are hosted on dbGaP at https://www.ncbi.nlm.nih.gov/projects/gap/cgi-bin/study.cgi?study_id=phs000774.v2.p1; while we cannot provide access to these protected data, any of our programming scripts may be made available upon request. All analyses were done in R version 4.4.1 [R Core Team, 2024].

S2 List of abbreviations

- GRM: genomic relatedness matrix
- GWAS: genome-wide association study
- LMM: linear mixed model
- PLMM: penalized linear mixed model
- POFC: Pittsburgh Orofacial Cleft Studies
- SNP: single nucleotide polymorphism

S3 Quality control procedures for PennCath data

The quality control steps implemented for the PennCath data were as follows:

1. All variants with missing call rates exceeding 0.1 were excluded from analysis.
2. All variants which had a Hardy-Weinberg equilibrium exact test p-value below 1e-10 were excluded from analysis.
3. Variants with a minor allele frequency (MAF) below 0.01 were excluded from analysis.
4. Samples with missing call rates exceeding 0.1 were excluded from the analysis.

All quality control (QC) was done in PLINK v. 1.9 [Purcell et al., 2007].

S4 Quality control procedures for POFC data

Details about QC for the samples:

- raw PLINK data: $N = 11,855$
- 2 samples removed for high degree of missingess ($>$ 0.05 of variants missing)
- 3 samples removed for sex discrepancies
- 1,305 samples removed due to not having complete data in corresponding phenotype file
- analytical sample: $N = 10,545$

Details about QC for variants:

- All variants with missing call rates exceeding 0.1 were excluded from analysis.
- All variants which had a Hardy-Weinberg equilibrium exact test p-value below 1e-10 were excluded from analysis. $512,926$ autosomal variants remained.
- The primary analysis of the POFC data was based on data from $469,577$ SNPs with minor allele frequency (MAF) above 0.0001.

S5 Cross-validation plots

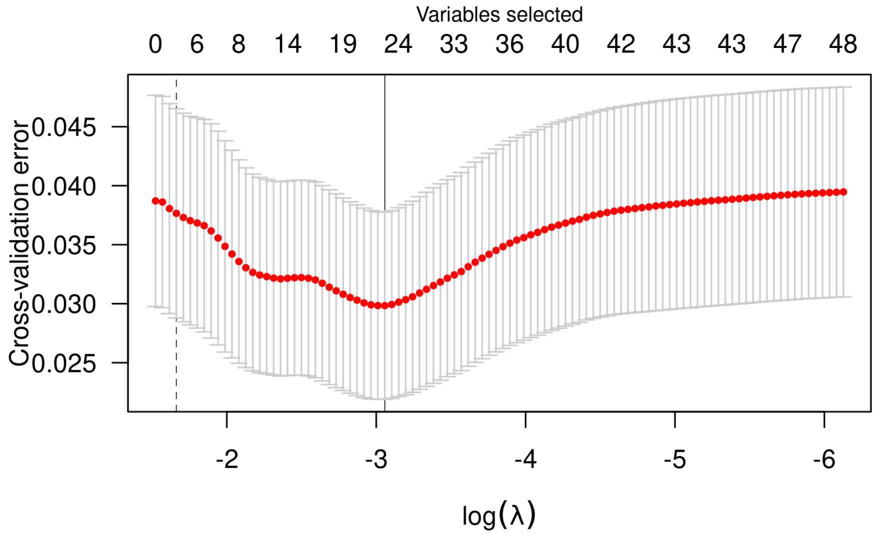

Figure S1: Plot of cross-validation error for **plmmr** models fit on the bladder cancer dataset ($n = 48$, $p = 22,283$). A sequence of 100 λ values was used in cross-validation.

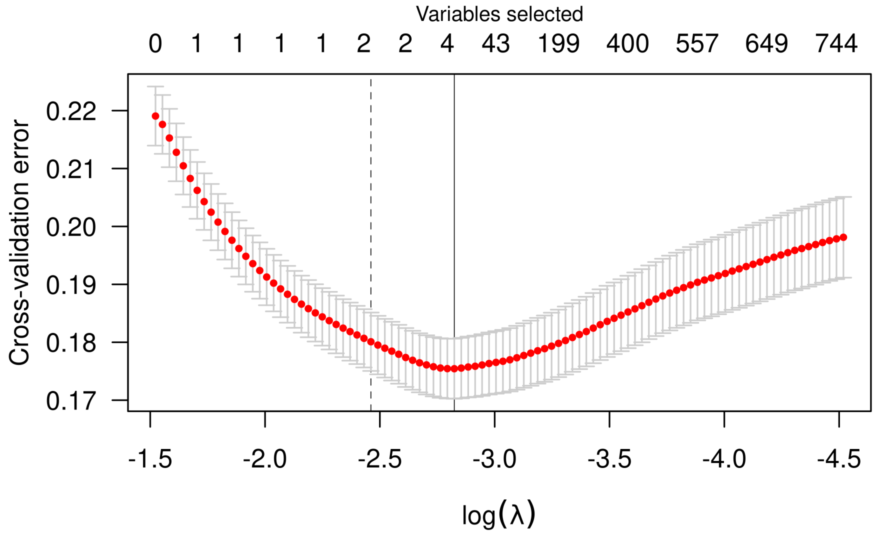

Figure S2: Plot of cross-validation error for **plmmr** models fit on the PennCath dataset ($n = 1,401$, $p = 696,646$). A sequence of 100 λ values was used in cross-validation.

S6 Computational considerations

Computational time and BLAS

While **plmmr** can analyze data sets of this size even with limited resources, we note that performance – particularly for operations such as the eigendecomposition of $\mathbf{K}$ – is substantially improved on systems that support multi-threaded BLAS.

Computing resources used in analysis of POFC data

For the analysis of the Pittsburgh Orofacial Cleft GWAS data, we used a high-performance computer in this case because of the size of n; in the current version of of **plmmr**, $\mathbf{X}$ is accessed via memory-mapping but $\mathbf{K}$ is held in-memory. Improving the scalability of **plmmr** for large n is an ongoing area of our work.

The **glmnet** package requires that data are stored in RAM, and so this analysis required a high-performance computer. We used the Interactive Data Analytics Service available through the University of Iowa for this task. The session in which our **glmnet** analysis of the POFC data was run had 16 available cores and 350 GiB of available RAM.

Computational considerations related to cross-validation

The **ggmix** and **PenalizedGLMM** packages rely on information criteria, namely the Akaike Information Criterion (AIC) and the Bayesian Information Criterion (BIC), for model selection. Although ggmix does not include a built-in cross-validation function, the relatively simplicity of implementing leave-one-out cross-validation manually made it a practical approach for comparing the performance of **ggmix** and **plmmr** on the bladder expression data. Given the developmental status of **PenalizedGLMM**'s cross-validation function and the complexities of implementing general cross-validation methods in the correlated data setting, we used information criteria for **PenalizedGLMM** model selection in the PennCath study analysis. The BIC was chosen for its tendency to select sparser models.

References

Shaun Purcell, Benjamin Neale, Kathe Todd-Brown, Lori Thomas, Manuel A. R. Ferreira, David Bender, Julian Maller, Pamela Sklar, Paul I. W. De Bakker, Mark J. Daly, et al. Plink: a tool set for whole-genome association and population-based linkage analyses. *The American journal of human genetics*, 81(3):559–575, 2007.

R Core Team. *R: A Language and Environment for Statistical Computing*. R Foundation for Statistical Computing, Vienna, Austria, 2024. URL https://www.R-project.org/.
